# Supplementary material for: Single Cell Transcriptomic and Chromatin Profiles Suggest Layer Vb Is the Only Layer With Shared Excitatory Cell Types in the Medial and Lateral Entorhinal Cortex
Source: Front Neural Circuits. 2022 Jan 26;15:806154. doi: 10.3389/fncir.2021.806154 (PMC8826650; doi:10.3389/fncir.2021.806154)
Supplement: Supplementary file 14 [file Data_Sheet_1.ZIP › LEC_Sup_vs_MEC_Sup_Motifs/homerResults/motif6.similar.html]

motif6

## Information for motif6

G
A
T
C
A
G
T
C
G
C
T
A
A
C
G
T
C
T
A
G
T
C
A
G
G
T
A
C
C
G
T
A
C
G
T
A
A
G
T
C
  
Reverse Opposite:  

T
C
A
G
A
C
G
T
A
C
G
T
C
A
T
G
A
G
T
C
G
A
T
C
C
G
T
A
C
G
A
T
T
C
A
G
C
T
A
G
  

|  |  |
| --- | --- |
| p-value: | 1e-37 |
| log p-value: | -8.630e+01 |
| Information Content per bp: | 1.738 |
| Number of Target Sequences with motif | 1160.0 |
| Percentage of Target Sequences with motif | 8.70% |
| Number of Background Sequences with motif | 1175.5 |
| Percentage of Background Sequences with motif | 5.90% |
| Average Position of motif in Targets | 275.9 +/- 203.8bp |
| Average Position of motif in Background | 317.2 +/- 247.5bp |
| Strand Bias (log2 ratio + to - strand density) | -0.0 |
| Multiplicity (# of sites on avg that occur together) | 1.10 |
| Motif File: | file (matrix) reverse opposite |

### Similar de novo motifs found

|  |  |  |  |  |  |  |  |
| --- | --- | --- | --- | --- | --- | --- | --- |
| Rank | Match Score | Redundant Motif | P-value | log P-value | % of Targets | % of Background | Motif file |
| 1 | 0.942 | C A T G A G C T C G A T C A T G A G T C A G T C C G T A C G A T C T A G C T A G G T C A C T G A | 1e-31 | -73.510414 | 11.43% | 8.44% | motif file (matrix) |
